# Prevalence and risk factors of intestinal parasitism among schoolchildren in the municipality of Banfora, Burkina Faso

**DOI:** 10.1016/j.fawpar.2026.e00345

**Published:** 2026-06-08

**Authors:** Mamoudou Cissé, Arthur Diakourga Djibougou, Souleymane Gnissi, Abdoul Aziz Nassa, Alamissa Soulama, Seydou Nakanabo Diallo, Issaka Zongo, Somkéta Thomas Venceslas Sawadogo, Adama Zida, Ibrahim Sangaré, Sanata Bamba

**Affiliations:** a Institut Supérieur des Sciences de la Santé, Université Nazi BONI, 01 1091, Bobo-Dioulasso 01, Burkina Faso; b Centre MURAZ, Institut National de Santé Publique, 01 BP390, Bobo-Dioulasso 01, Burkina Faso; c Centre de Recherche en Santé de Nouna, Institut National de Santé Publique, BP 02, Nouna, Burkina Faso; d Institut de Recherche en Sciences de Santé, Centre National de la Recherche Scientifique et Technologique, 01 BP 545, Bobo-Dioulasso 01, Burkina Faso; e Unité de Formation et de Recherche en Sciences de la Santé, Université Joseph KI-ZERBO, 03 BP 7021, Ouagadougou 03, Burkina Faso

**Keywords:** Intestinal parasitism, Schoolchildren, Prevalence, Risk factors, Burkina Faso

## Abstract

Intestinal parasitic infections remain a major public health concern, particularly among school-aged children in resource-limited settings. This study aimed to determine the prevalence and risk factors of intestinal parasitism among schoolchildren in the municipality of Banfora, Burkina Faso.

A cross-sectional study was conducted in November 2024 among 298 schoolchildren aged 5–14 years. Sociodemographic, environmental, and behavioral data were collected using a structured questionnaire. Stool samples were examined by direct wet mount, formol-ether concentration, and Kato-Katz techniques. Factors associated with intestinal parasitism were identified using multivariable logistic regression.

The median age of the participants was 9 years (range: 5–13). The overall prevalence of intestinal parasitism was 81.9% (244/298; 95% CI: 77.3–86.2). Protozoa predominated (238/298, 79.9%), mainly *Entamoeba histolytica/dispar* (55.7%). Helminths accounted for 8.4% (25/298) of infections, with *Schistosoma mansoni* being the most frequent (5.7%). Low personal hygiene (adjusted OR = 2.2; 95% CI: 1.1–4.2) and household ownership of domestic animals (adjusted OR = 2.1; 95% CI: 1.1–4.1) were identified as the main risk factors for orally transmitted intestinal parasitism. In contrast, having a non-educated household head (adjusted OR = 3.7; 95% CI: 1.1–12.8) was the primary risk factor for skin-transmitted pathogenic intestinal parasitic infections.

Intestinal parasitism is highly endemic among schoolchildren in Banfora. Strengthening integrated control strategies combining hygiene promotion, sanitation improvement, health education, and deworming is essential to sustainably reduce the burden of intestinal parasitism.

## Highlights

• Intestinal parasitic infections affected 81.9% of schoolchildren in Banfora.
• Protozoan infections predominated, led by *Entamoeba histolytica/dispar*.
• *Schistosoma mansoni* was the most common helminth species detected.
• Poor hygiene and animal ownership increased infection risk.
• Integrated control measures are needed to reduce parasite burden.

## 1 Introduction

Intestinal parasitic infections (IPIs) remain a major global public health problem, affecting more than one billion people worldwide, particularly in tropical and low-resource settings (Ahmed, 2023; Chen et al., 2024; Torgerson et al., 2015). Their persistence is strongly associated with poverty, limited access to safe drinking water, poor sanitation, and inadequate hygiene practices, which facilitate fecal–oral transmission (Ahmed, 2023; WHO, 2023b).

In sub-Saharan Africa, geohelminth infections and schistosomiasis due to *Schistosoma (S.) mansoni* are highly prevalent. Protozoan parasites, such as *Giardia (G.) intestinalis*, *Entamoeba (E.) histolytica/dispar*, and *Cryptosporidium* spp. are also commonly reported (Ahmed, 2023; Torgerson et al., 2015; WHO, 2023a). School-aged children are particularly vulnerable, with an estimated 654 million children requiring preventive or therapeutic interventions (WHO, 2023a). Chronic infections are associated with anaemia, cognitive impairment, malnutrition, and poor school performance (Ahmed, 2023; King and Dangerfield-Cha, 2008; WHO, 2023a, WHO, 2023b).

Higher prevalences of IPIs have been reported across various African settings, ranging from 70.5% to 95.3% (Gyang et al., 2019; Irisarri-Gutiérrez et al., 2022; Njambi et al., 2020). In addition, intestinal protozoan parasites affect approximately 25% of schoolchildren in Africa, although prevalence varies considerably across regions (Hajissa et al., 2022). Reported risk factors for IPIs include age, water and sanitation conditions, hygiene practices, presence of domestic animals, and household socioeconomic status (SES) (Erismann et al., 2016; Gupta et al., 2020; Kesete et al., 2020; Muadica et al., 2021; Shrestha et al., 2021; Yin et al., 2022). These determinants underscore the need for integrated control strategies, combining water and sanitation improvements, health education, behavioral change, and regular deworming.

In Burkina Faso, IPIs remain a significant public health concern, especially among schoolchildren. Since the early 2000s, mass deworming campaigns have been implemented in accordance with World Health Organization (WHO) recommendations, alongside interventions targeting hygiene, sanitation, and access to safe water (Drabo et al., 2016; Ouedraogo et al., 2016). Despite a substantial reduction in helminth infections, intestinal parasitism remains common. In 2024, it accounted for 1,041,174 outpatient consultations, ranking as the sixth leading cause of consultation, including 215,553 children aged 5–14 years (Ministère de la santé, 2025).

However, data on the prevalence and risk factors of intestinal parasitism among schoolchildren in Burkina Faso remain limited and heterogeneous, with reported prevalences ranging from 26.2% to 84.7% (Erismann et al., 2016; Ouoba, 2017; Soma, 2025). Some regions, including the Cascades region, are particularly understudied, despite environmental conditions favorable to transmission, such as the Comoé River, ponds, lakes, and lowland rice fields.

Therefore, this study aimed to assess the prevalence of intestinal parasitism and to identify associated factors among schoolchildren in the commune of Banfora, to generate updated evidence to support the evaluation and optimization of prevention and control strategies.

## 2 Materials and methods

### 2.1 Study design and setting

A cross-sectional study was conducted in November 2024 in the municipality of Banfora, Cascades region, southwestern Burkina Faso. The study area has been described in detail elsewhere (Cissé et al., 2025). The survey was carried out in five public primary schools located within 500 m of surface water sources, including two urban and three rural schools.

### 2.2 Study population

The study population consisted of schoolchildren enrolled in the selected schools during the 2024–2025 academic year. Children were eligible if they were aged 4–15 years, had resided in Banfora for at least six months, and had written informed consent provided by a parent or legal guardian. Assent was obtained from children aged ≥12 years.

Children were excluded if they were absent on the day of data or sample collection, unable to provide a stool sample, severely ill at the time of the survey, or had received antiparasitic treatment within the six months preceding the study.

### 2.3 Sample size and sampling procedure

Sample size was determined according to the WHO recommendations, which advise a minimum of 50 schoolchildren per school for baseline parasitological surveys (WHO, 2006). For practical considerations, 60 children per school were included, yielding a final sample size of 300 participants.

Within each school, simple random sampling was applied. Class registers were used as sampling frames, and 10 children per class were randomly selected until the required sample size was reached.

### 2.4 Field data and stool samples collection

Following the informed consent and assent procedures, field data were collected through face-to-face interviews with schoolchildren and their parents or legal guardians using a structured questionnaire. The questionnaire captured information on sociodemographic characteristics, household conditions, and environmental and behavioral exposure factors related to intestinal parasitism.

Each participating child received a clean, labeled stool container with instructions for sample collection. Stool samples were collected on the same day or the following morning and transported to the laboratory in a cool box. Initial processing for the detection of protozoan trophozoites and helminth larvae was performed at the Centre Médical Urbain of Banfora. Samples were subsequently stored at −20 °C and transported under cold conditions to the Parasitology–Mycology Laboratory of Centre MURAZ for the detection of protozoan cysts and helminth eggs.

### 2.5 Laboratory procedures

Stool samples were analyzed for intestinal parasites using a combination of direct wet mount microscopy, the Kato-Katz method, and the formol-ether concentration technique, in accordance with standard parasitological protocols. All laboratory analyses were performed by trained personnel in compliance with established standard operating procedures to ensure the accuracy and reliability of the results.

#### 2.5.1 Direct wet mount examination

Fresh stool samples (approximately 2 mg) were placed on a microscope slide using a wooden applicator, emulsified with a drop of physiological saline (0.9%), covered with a coverslip, and examined under a light microscope at 10× and 40× magnifications, as previously described (Yimer et al., 2015). This method was primarily used for the detection of motile protozoan trophozoites, as well as cysts, helminth eggs, and larvae.

#### 2.5.2 The formol-ether concentration technique

This method was used to enhance the detection of protozoan cysts and helminth eggs, particularly in samples with low parasite density. Approximately 2 g of stool were emulsified in 7 mL of 10% formalin solution, filtered to remove large debris, and mixed with 3 mL of ether. The suspension was vigorously shaken for 30 s and centrifuged at 2000 rpm for 2 min. After discarding the supernatant, the sediment was mounted on a microscope slide, stained with Lugol's iodine, covered with a coverslip, and examined at 10× and 40× magnifications, as previously described (Ritchie, 1948).

#### 2.5.3 The Kato-Katz technique

This technique was performed according to WHO guidelines. For each stool sample, one Kato-Katz thick smear was prepared using the standard 41.7 mg template and was examined independently by two trained laboratory biologists. Slides were allowed to clear for 24 h prior to examination to facilitate the visualization of *S. mansoni* and soil-transmitted helminths (STH) eggs (Katz et al., 1972). The intensity of *S. mansoni* and STH infection was estimated by multiplying the total number of eggs counted by 24, with results expressed as eggs per gram of feces (EPG). Infection intensity was categorized based on WHO thresholds (Montresor et al., 1998).

### 2.6 Statistical analysis

Data were entered twice independently using Epi Info version 7.2.6.0 and exported to Microsoft Excel 2019. Data cleaning and statistical analyses were performed using Stata version 12 (StataCorp, College Station, TX, USA).

Categorical variables were summarized as frequencies and percentages, whereas continuous variables were described using medians and interquartile ranges (IQR) after assessment of data distribution.

A stool sample was considered positive if at least one intestinal parasite was detected by any of the diagnostic methods employed. The prevalence of intestinal parasitism was calculated with corresponding 95% confidence intervals (95% CI).

Household socioeconomic status (SES) was derived using principal component analysis (PCA) based on household and housing characteristics, including housing construction materials (walls, roof, and floor), source of drinking water, sanitation facilities, primary energy source, and ownership of domestic animals. A composite wealth score was generated and categorized into tertiles (low, moderate, and high SES).

The level of personal hygiene among schoolchildren was assessed using PCA as part of the Hygiene component of the Water, Sanitation and Hygiene (WASH) framework. The analysis was based on hygiene-related variables, including nail trimming status, handwashing before and after meals, handwashing after defecation, handwashing after playing, and the method of handwashing (water only or water with soap). A composite hygiene score was generated and categorized into tertiles (low, moderate, and high).

Associations between intestinal parasitism (dependent variable) and explanatory variables were assessed using logistic regression analysis. All independent variables with a *p*-value <0.20 in the univariate analysis were entered into a multivariable logistic regression model. An automatic backward stepwise selection procedure was applied, whereby variables were sequentially removed based on the Wald test, using an exclusion criterion of *p* > 0.05. Only variables that remained statistically significant at *p* < 0.05 were retained in the final model. The goodness-of-fit and specification of the final model were evaluated.

The strength of associations was expressed as adjusted odds ratios (aORs) with their corresponding 95% confidence intervals (95% CIs). A p-value <0.05 was considered statistically significant.

### 2.7 Ethical considerations

The study received ethical approval from the Comité d'éthique institutionnel de l'Institut National de Santé Publique du Burkina Faso (Approval No. 2024–0011/MSHP/SG/INSP/DG/CEI). Written informed consent was obtained from parents or legal guardians, and assent was also obtained from children aged ≥12 years. Data confidentiality was ensured through anonymization. All children diagnosed with IPIs received free treatment according to national guidelines.

## 3 Results

### 3.1 Characteristics of the study population

A total of 298 out of the 300 initially selected schoolchildren were included in the study, as stool samples could not be obtained from two participants. Although the eligibility criteria covered ages 4–15 years, the final sample included children aged 5–13 years, corresponding to those who were enrolled and provided complete data. The median age of the included children was 9 years (range: 5–13 years). Sex distribution was balanced, with a slight predominance of girls (51.3%). Most children lived in rural areas (61.7%), and 75.5% were under 10 years old. Agriculture was the main occupation of 76.2% of household heads. Household size ranged from 3 to 23 persons (median: 9; IQR = 6). Educational attainment was low, with 63.4% of household heads and 77.1% of mothers being illiterate. Overall, 39.3% of households had a low SES and 31.5% a moderate status. Most households (72.5%) were located within 500 m of a water source, but only 5% had piped water; drinking water mainly came from boreholes (72.8%) and traditional wells (22.1%). Regarding sanitation, 8.1% of households had no latrine, 50.3% used traditional latrines, and 38.9% used improved latrines. Ownership of domestic animals was reported in 82.2% of households (Table 1).

**Table 1:** Characteristics of the study population.

Variables	Frequency	%
Age group (years)		
5–10	225	75.5
˃ 10	73	24.5
Sex		
Female	153	51.3
Male	145	48.7
Residence		
Rural	184	61.7
Urban	114	38.3
Main occupation of the head of household		
Agriculture	227	76.2
Trader	43	14.4
Civil servant	16	5.4
Others (retirement and no employment)	12	4.0
Educational level of the household head^a^		
No formal schooling	187	63.4
Primary education	61	20.7
Secondary or higher education	47	15.9
Educational level of the schoolchild's mother^b^		
No formal schooling	229	77.1
Primary education	32	10.8
Secondary education	36	12.2
Household ownership of domestic animals		
Yes	245	82.2
No	53	17.8
Household size		
≤ 9 individuals	133	44.6
˃ 9 individuals	165	55.4
Household source of drinking water		
Well	66	22.2
Borehole	217	72.8
Piped water	15	5.0
Household sanitation facility		
No latrine	24	8.1
Traditional latrine	150	50.3
Improved latrine	116	38.9
Modern latrine	8	2.7
Household SES		
Low	117	39.3
Moderate	94	31.5
High	87	29.2
Distance from the household to the water point		
≤ 500 m	216	72.5
> 500 m	82	27.5

SES: Socioeconomic status.

a 3 missing data.

b 1 missing data.

### 3.2 WASH conditions and behavioral factors among schoolchildren

Among schoolchildren, 18.5% had a high level of personal hygiene, while 34.2% had a low hygiene level. The main sources of drinking water were boreholes (70.8%) and wells (20.5%). Latrine non-use was reported by 10.4% of children. Most children did not wear shoes (61.1%) and did not regularly visit the village water source (73.8%) (Table 2).

**Table 2:** WASH conditions and behavioral factors among schoolchildren.

Variables	Frequency	%
Source of drinking water		
Well	26	8.7
Borehole	211	70.8
Piped water	61	20.5
Level of personal hygiene		
Low	102	34.2
Moderate	141	47.3
High	55	18.5
Toilet use frequency		
None	31	10.4
Occasional	148	49.7
Frequent	119	39.9
Footwear use		
Yes	116	38.9
No	182	61.1
Go to the water point		
Yes	78	26.2
No	220	73.8

### 3.3 Prevalence of intestinal parasitism

Among the 298 stool samples analyzed, 244 were positive for at least one intestinal parasite, yielding an overall prevalence of 81.9% (95% CI: 77.3–86.2). Polyparasitism was common, with up to four parasite species identified in a single host. Among the 244 infected children, 168 (68.8%) harbored a single parasite species, 57 (23.4%) had dual infections, 17 (7.0%) had triple infections, and 2 (0.8%) presented with quadruple infections. More than half of the dual infections (52.6%, 30/57) consisted of the co-occurrence of *E. coli* and *E. histolytica/dispar*.

**Fig. 1:**
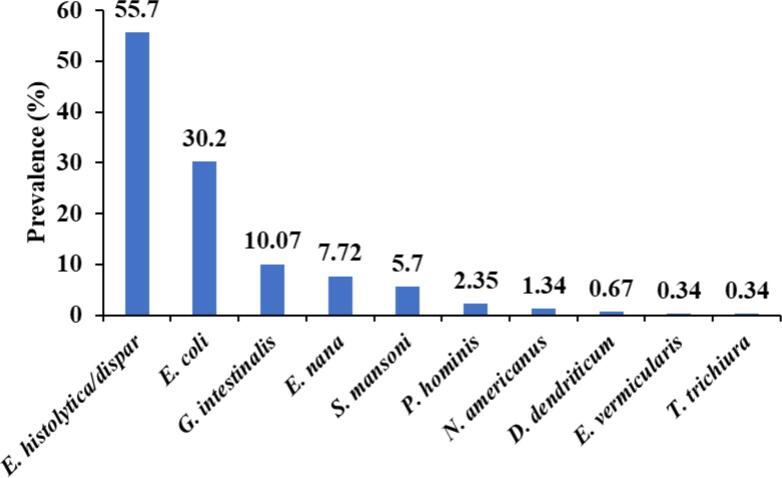
Distribution of intestinal parasite species among schoolchildren. *E. histolytica*/dispar: *Entamoeba histolytica*/dispar; *E. coli*: *Entamoeba coli*; *G. intestinalis*: Giardia intestinalis; *E. nana*: *Endolimax nana*; *S. mansoni*: *Schistosoma mansoni*; *P. hominis*: Pentatrichomonas hominis; *N. americanus*: *Necator americanus*; *D. dendriticum*: *Dicrocoelium dendriticum*; *E. vermicularis*: *Enterobius vermicularis*; T. trichiura: Trichuris trichiura.

Protozoa predominated with 238 cases (79.9%; 95% CI: 75.3–84.4), whereas helminths were detected in 25 samples (8.4%; 95% CI: 5.2–11.6). The highest prevalence was observed using direct wet mount examination (74.2%, 221/298), followed by the formol-ether concentration technique (29.5%, 88/298) and the Kato-Katz method (5.7%, 17/298). Among protozoa, *E. histolytica/dispar* was the most prevalent (55.7%), followed by *E. coli* (30.2%) and *G. intestinalis* (10.1%). Among helminths, *S. mansoni* was the predominant species (5.7%) (Fig. 1).

### 3.4 Factors associated with orally transmitted intestinal parasitism

Parasites classified as orally transmitted intestinal parasitism included *E. coli*, *E. histolytica/dispar*, *G. intestinalis*, *Pentatrichomonas hominis*, *Enterobius (E.) vermicularis*, *Trichuris trichiura*, and *Dicrocoelium (D.) dendriticum*. In univariate analysis, low personal hygiene was significantly associated with a higher risk of orally transmitted intestinal parasitism (crude OR = 2.8; 95% CI: 1.2–6.6). Children from households with moderate (crude OR = 2.2; 95% CI: 1.1–4.6) and low (crude OR = 2.3; 95% CI: 1.2–4.6) SES also had an increased risk. Additionally, household ownership of domestic animals was associated with higher odds of oral transmission of intestinal parasitism (crude OR = 2.8; 95% CI: 1.2–4.4).

In multivariable analysis, low personal hygiene remained significantly associated with higher odds of orally transmitted intestinal parasitism (adjusted OR = 2.2; 95% CI: 1.1–4.2). Similarly, children living in households owning domestic animals had approximately twice the risk compared with those without animals (adjusted OR = 2.1; 95% CI: 1.1–4.1) (Table 3).

**Table 3:** Univariate and multivariable analysis of factors associated with orally transmitted intestinal parasitism. Bold values indicate statistically significant associations with intestinal parasitism (p < 0.05).

Variables	Orally transmitted intestinal parasitism		COR (95% CI)	*P*-value	AOR (95% CI)	*P*-value
	Yes (%)	No (%)				
Age group (years)						
5–10	181 (80.4)	44 (19.6)	1.1 (0.6–2.1)	0.85		
˃ 10	58 (79.5)	15 (20.5)	1			
Sex						
Female	127 (83.1)	26 (17.0)	1.4 (0.8–2.6)	0.21		
Male	112 (77.2)	33 (22.8)	1			
Residence						
Rural	151 (82.1)	33 (17.9)	1.4 (0.8–2.4)	0.31		
Urban	88 (77.2)	26 (22.81)	1			
Level of personal hygiene						
Low	90 (88.2)	12 (11.8)	**2.8 (1.2–6.6)**	**0.02**	**2.2 (1.1–4.2)**	**0.02**
Moderate	109 (77.3)	32 (22.7)	1.3 (0.6–2.6)	0.50	–	–
High	40 (72.7)	15 (27.3)	1		1	
Source of drinking water						
Well	23 (88.5)	3 (11.5)	3.0 (0.8–11.2)	0.11	–	–
Borehole	172 (81.5)	39 (18.5)	1.7 (0.9–3.3)	0.11	–	–
Piped water	44 (72.1)	17 (27.9)	1			
Use of toilet						
Yes	210 (78.7)	57 (21.4)	1			
No	29 (93.6)	2 (6.5)	3.9 (0.9–17.0)	0.07	–	–
Educated household head						
Yes	84 (77.1)	25 (22.9)	1			
No	155 (82.1)	34 (18.0)	1.4 (0.8–2.4)	0.30		
Educated mother						
Yes	51 (75.0)	17 (25.0)	1			
No	187 (81.7)	42 (18.3)	1.5 (0.8–2.8)	0.23		
Household size						
≤ 9 individuals	102 (76.7)	31 (23.3)	1			
˃ 9 individuals	137 (83.0)	28 (17.0)	1.5 (0.8–2.6)	0.17	–	–
Household SES						
High	61 (70.1)	26 (29.9)	1			
Moderate	79 (84.0)	15 (16.0)	**2.2 (1.1–4.6)**	**0.03**	–	–
Low	99 (84.6)	18 (15.4)	**2.3 (1.2–4.6)**	**0.01**	–	–
Household source of drinking water						
Well	12 (80.0)	3 (20.0)	1.5 (0.4–5.9)	0.56	–	–
Borehole	179 (82.5)	38 (17.5)	1.8 (0.9–3.4)	0.08	–	–
Piped water	48 (72.7)	18 (27.3)	1			
Household ownership of a sanitation facility						
No	20 (83.3)	4 (16.7)	1			
Yes	219 (79.9)	55 (20.1)	0.8 (0.3–2.4)	0.69		
Household ownership of domestic animals						
Yes	203 (82.9)	42 (17.1)	**2.28 (1.2–4.4)**	**0.02**	**2.1 (1.1–4.1)**	**0.03**
No	36 (67.9)	17 (32.1)	1			
Distance from the household to the water point						
≤ 500 m	171 (79.2)	45 (20.8)	1			
> 500 m	68 (82.9)	14 (17.1)	1.3 (0.7–2.5)	0.47		

AOR: adjusted odds ratio; COR: crude odds ratio; SES: socioeconomic status.

### 3.5 Factors associated with skin-transmitted IPIs

Skin-transmitted intestinal parasites included *S. mansoni* and *Necator americanus*. Univariate analysis revealed a significant association between the educational status of the household head and skin-transmitted IPIs (crude OR = 3.7; 95% CI: 1.1–12.9). In multivariable analysis (Table 4), having a non-educated household head (adjusted OR = 3.7; 95% CI: 1.1–12.8) was identified as the main risk factor for skin-transmitted IPIs.

**Table 4:** Univariate and multivariable analysis of factors associated with skin-transmitted IPIs. Bold values indicate statistically significant associations with intestinal parasitism (p < 0.05).

Variables	Skin-transmitted IPIs		COR (95% CI)	*P*-value	AOR (95% CI)	*P*-value
	Yes (%)	No (%)				
Age group (years)						
5–10	17 (7.6)	208 (92.4)	1.4 (0.5–4.3)	0.55		
˃ 10	4 (5.5)	69 (94.5)	1			
Sex						
Female	11 (7.2)	142 (92.8)	1.1 (0.4–2.5)	0.92		
Male	10 (6.9)	135 (93.1)	1			
Residence						
Rural	12 (6.5)	172 (93.5)	1			
Urban	9 (7.9)	105 (92.1)	1.2 (0.5–3.0)	0.65		
Footwear use						
Yes	7 (6.0)	109 (94.0)	1			
No	14 (7.7)	168 (92.3)	1.3 (0.5–3.3)	0.59		
Go to the water point						
Yes	13 (5.9)	207 (94.1)	1			
No	8 (10.3)	70 (89.7)	0.6 (0.2–1.4)	0.20		
Use of toilet						
Yes	17 (6.4)	250 (93.6)	1			
No	4 (12.9)	27 (87.1)	2.2 (0.7–6.9)	0.19	–	–
Educated household head						
Yes	3 (2.8)	106 (97.2)	1			
No	18 (9.5)	171 (90.5)	**3.7 (1.1–12.9)**	**0.04**	**3.7 (1.1–12.8)**	**0.04**
Educated mother						
Yes	2 (2.9)	66 (97.1)	1			
No	19 (8.3)	210 (91.7)	3.0 (0.7–13.2)	0.15	–	–
Household size						
≤ 9 individuals	11 (8.3)	122 (91.7)	1			
˃ 9 individuals	10 (6.1)	155 (93.9)	0.7 (0.3–1.7)	0.46		
Household SES						
High	8 (9.2)	79 (90.8)	1			
Moderate	6 (6.4)	88 (93.6)	0.7 (0.2–2.0)	0.48		
Low	7 (6.0)	110 (94.0)	0.6 (0.2–1.8)	0.39		
Household ownership of a sanitation facility						
Yes	20 (7.3)	254 (92.7)	1.8 (0.2–14.1)	0.57		
No	1 (4.2)	23 (95.4)	1			
Distance from the household to the water point						
≤ 500 m	15 (6.9)	201 (93.1)	1			
> 500 m	6 (7.3)	76 (92.7)	1.1 (0.4–2.8)	0.91		

AOR: adjusted odds ratio; COR: crude odds ratio; IPIs: intestinal parasitic infections; SES: socioeconomic status.

## 4 Discussion

The overall prevalence of intestinal parasitism in this study (81.9%) indicates a high level of endemicity among schoolchildren in the study area. This prevalence is comparable to that reported in other studies conducted in Burkina Faso and elsewhere in Africa. At the national level, similar prevalences were reported in the Plateau Central and Centre-Ouest regions (84.7%) (Erismann et al., 2016) and in Yamtenga, Koubri, and Daguilma (75%) (Savadogo et al., 2015). In contrast, lower prevalences were reported in other settings of the country, including Oubritenga Province (26.2%) (Soma, 2025), the rural municipalities of Coalla and Manni (57.6%) (Ouoba, 2017), and Bobo-Dioulasso (40.1%) (Zongo, 2003). These differences suggest a heterogeneous distribution of intestinal parasitism across Burkina Faso, likely related to sociodemographic characteristics, local environmental conditions, and regional differences in control interventions.

Higher prevalences have been reported in other African settings, such as Nigeria (86.2%) (Gyang et al., 2019) and Rwanda (95.3%) (Irisarri-Gutiérrez et al., 2022), while comparatively lower rates within Africa have been observed in Kenya (70.5%)(Njambi et al., 2020). In contrast, studies conducted outside Africa, including in Nepal (33%) (Gupta et al., 2020) and Colombia (29.6%) (Quiroz et al., 2020), have reported even lower prevalences. Such variability may reflect differences in ecological settings, socioeconomic conditions, diagnostic approaches, and the coverage and effectiveness of parasite control programs.

It is important to note that the overall prevalence of intestinal parasitism reported in this study includes non-pathogenic species such as *E. coli*, *Endolimax nana*, and *P. hominis*. Although these organisms are not associated with clinical disease, their presence is indicative of fecal–oral exposure and poor hygiene conditions. Their inclusion may have contributed to the high prevalence observed and should be considered when comparing results with studies that report only pathogenic parasites. Nonetheless, these species remain epidemiologically relevant as markers of environmental contamination and transmission risk.

The parasitic profile observed in this study was dominated by intestinal protozoa (79.9%), indicating a predominantly fecal–oral transmission, likely driven by contaminated water and food, as well as poor hygiene practices. The predominance of protozoa, which are less susceptible to mass drug administration (MDA), suggests the persistence of environmental and behavioral risk factors. Similar patterns have been reported in Burkina Faso (Erismann et al., 2016; Ouoba, 2017; Soma, 2025), other parts of Africa (Irisarri-Gutiérrez et al., 2022; Muadica et al., 2021), and Asia (Gupta et al., 2020; Al-Fakih et al., 2022), confirming the widespread distribution of this profile across diverse settings.

The low prevalence of helminths (8.4%) suggests reduced transmission, likely reflecting the impact of school-based MDA programs, as previously reported in Burkina Faso (Drabo et al., 2016; Ouedraogo et al., 2016; Zongo et al., 2025). In contrast, helminth-dominated profiles reported in Ghana (Donkoh et al., 2023), Ethiopia (Sitotaw and Shiferaw, 2020), Kenya (Njambi et al., 2020), and Colombia (Quiroz et al., 2020) highlight the influence of local environmental conditions and variations in control program coverage. These findings highlight the need to sustain therapeutic interventions while strengthening WASH measures to ensure long-term control and prevent helminth resurgence. Although biologically plausible, the impact of current WASH interventions has often been lower than expected, likely due to limitations in implementation and uptake (Garn et al., 2022). This underscores the need for more targeted implementation research and better standardization of infection metrics.

*E. histolytica/dispar* was the most prevalent protozoan (55.7%), suggesting active fecal-oral transmission, likely driven by untreated water consumption, inadequate hand hygiene, and contaminated food. This finding reflects suboptimal hygiene and sanitation conditions and underscores the need to improve access to safe drinking water, environmental sanitation, and health education.

The prevalence observed in our study was lower than that reported by Erismann et al. in Burkina Faso (66.5%) (Erismann et al., 2016), but higher than those reported in other settings within Burkina Faso (0.8% (Soma, 2025) and 31.52% (Ouoba, 2017)) and elsewhere in Africa (Aschale et al., 2021; Hailu and Alemu, 2024; Sitotaw and Shiferaw, 2020). These differences may be attributed to variations in hygiene and sanitation conditions, environmental factors affecting cyst survival, and diagnostic approaches, as conventional microscopy cannot differentiate *E. histolytica* from *E. dispar*, potentially leading to overestimation of prevalence.

*S. mansoni* was the most prevalent helminth (5.7%). This finding is notable, as *S. mansoni* schistosomiasis had been considered absent from the study area since 1978 (3.7%) (Boudin and Simonkovich, 1978), suggesting either re-emergence of transmission linked to environmental exposure to freshwater bodies, or that past surveys underestimated its presence. These results highlight the need to strengthen surveillance, maintain praziquantel-based control interventions, and implement targeted prevention measures. The prevalence observed in this study was higher than previous reports from Burkina Faso, including the 0.3% reported in the Plateau Central and Centre-Ouest regions (Erismann et al., 2016) and the recent national estimate of 0.1% (Zongo et al., 2025). It was comparable to the prevalence reported in Ethiopia (4.4%) (Sitotaw and Shiferaw, 2020) but markedly lower than that reported in Kenya (70.5%) (Njambi et al., 2020). This variability likely reflects differences in environmental conditions, coverage and effectiveness of control programs, and population behaviors involving contact with water bodies (Njambi et al., 2020).

Low personal hygiene was identified as the main risk factor for orally transmitted intestinal parasitism, consistent with the fecal–oral route of transmission (ANOFEL, 2021). Previous studies in Africa have highlighted nail trimming, handwashing, and soap use as critical behaviors (Aschale et al., 2021; Belete et al., 2021; Sitotaw and Shiferaw, 2020). These findings underscore the need to prioritize school-based hygiene promotion, including regular handwashing with soap, proper nail care, and targeted health education. Combined with improved sanitation and deworming programs, such measures are essential for sustainably reducing transmission (Garn et al., 2022).

Schoolchildren living in households owning domestic animals had an approximately twofold increased risk of orally transmitted intestinal parasitism, suggesting that animals may act as reservoirs or indirect sources of contamination, particularly for fecal-oral transmission. These findings are consistent with studies conducted in Burkina Faso (Erismann et al., 2016), Mozambique (Muadica et al., 2021), and Nepal (Shrestha et al., 2018), but contrast with results from Yemen (Al-Fakih et al., 2022), indicating that the impact of domestic animal ownership may vary across environmental and behavioral contexts. These results support the need for an integrated One Health approach combining hygiene promotion and improved management of domestic animals to reduce transmission.

Schoolchildren living in households headed by non-educated individuals had an approximately twofold higher risk of skin-transmitted IPIs. Similar associations have been reported in some settings (Feleke et al., 2022), whereas no significant relationship was observed elsewhere (Alemu et al., 2016; Sitotaw and Shiferaw, 2020), highlighting context-dependent variability. This association suggests that household head education may influence children's exposure through preventive knowledge and practices, including shoe wearing and avoidance of contaminated soil. These findings underscore the need to strengthen family-centered health education and promote protective behaviors, alongside improved access to deworming and preventive interventions, to reduce transmission.

This study has several limitations. First, its restriction to a specific geographical setting and the selection of schools within 500 m of surface water sources may limit the generalizability of the findings and introduce a bias toward higher-risk environments. Second, the cross-sectional design precludes establishing temporal relationships between exposures and outcomes. Methodological and resource constraints may also have affected diagnostic accuracy. The use of a single stool sample may have reduced sensitivity, particularly for *G. intestinalis*, while the absence of Graham's adhesive tape technique may have led to underdetection of *E. vermicularis*. Additionally, microscopy does not allow differentiation between *E. histolytica* and *E. dispar*, potentially overestimating pathogenic infections. The lack of a liver-free diet protocol also prevents distinguishing true infection from pseudoparasitosis for *D. dendriticum*. Furthermore, the inclusion of non-pathogenic species such as *E. coli*, *E. nana*, and *P. hominis* in the analysis may have influenced overall prevalence estimates and should be interpreted as indicators of intestinal parasitism and fecal exposure rather than true infection burden. Finally, self-reported behavioral data may be subject to reporting bias.

## 5 Conclusion

The present study reveals a very high prevalence of intestinal parasitism among schoolchildren in Banfora, confirming their persistence as a major public health concern. The diversity of parasites identified indicates ongoing transmission, driven by environmental and socioeconomic factors, with potential impacts on child health and academic performance. Low personal hygiene, domestic animal ownership, and low educational level of the household head were significantly associated factors. These findings underscore the need for integrated control strategies combining health education, improved hygiene and sanitation, and continued deworming, alongside longitudinal studies to guide sustainable interventions.

## CRediT authorship contribution statement

**Mamoudou Cissé:** Writing – review & editing, Writing – original draft, Visualization, Validation, Supervision, Project administration, Methodology, Investigation, Funding acquisition, Formal analysis, Data curation, Conceptualization. **Arthur Diakourga Djibougou:** Writing – review & editing, Methodology, Investigation. **Souleymane Gnissi:** Writing – review & editing, Investigation. **Abdoul Aziz Nassa:** Writing – review & editing, Investigation. **Alamissa Soulama:** Writing – review & editing, Supervision, Methodology, Investigation. **Seydou Nakanabo Diallo:** Writing – review & editing, Methodology. **Issaka Zongo:** Writing – review & editing, Methodology. **Somkéta Thomas Venceslas Sawadogo:** Writing – review & editing. **Adama Zida:** Writing – review & editing. **Ibrahim Sangaré:** Writing – review & editing. **Sanata Bamba:** Writing – review & editing.

## Ethical approval and consent to participate

This study was approved by the Comité d'éthique institutionnel de l'Institut National de Santé Publique (approval number 2024–0011/MSHP/SG/INSP/DG/CEI). Written informed consent was obtained from all study participants.

## Funding

Field data collection and lab work were supported by funds from a 10.13039/100004421World Bank African Centres of Excellence grant (ACE02-WACCBIP) as part of a regional partnership between Centre MURAZ and the University of Ghana. The funder had no role in study design, data collection and analysis, decision to publish, or preparation of the manuscript.

## Declaration of competing interest

The authors declare that they have no competing interests.

## Data Availability

Data is contained within the article.
